# A teenage girl with an untreatable nephrotic syndrome: Questions

**DOI:** 10.1007/s00467-020-04706-0

**Published:** 2020-07-27

**Authors:** Marta Cognigni, Marco Pennesi, Giulia Pennesi, Egidio Barbi

**Affiliations:** 1 University of Trieste, Trieste, Italy; 2 Institute for Maternal and Child Health—IRCCS Burlo Garofolo, Trieste, Italy; 3 Edinburgh Napier University, Edinburgh, UK

## Case

A 17-year-old girl was referred to our care due to periorbital and lower limb oedema, high fever and asthenia that had been persisting for over a week. The urine dipstick tested positive for proteins (4+). Urine analysis detected high levels in nephrotic range (1873 mg 24 h) and blood tests showed hypoalbuminaemia (1.1 g/dL), hyperlipidaemia (cholesterol 333 mg/dL), hyperfibrinogenemia (1394 mg/dL) and high C-reactive protein (CRP 7.5 mg/dL). A nephrotic syndrome (NS) was therefore diagnosed.

Abdominal and cardiac ultrasound, kidney Doppler study, chest X-ray and ophthalmologic evaluation, performed to rule out a TINU (tubulointerstitial nephritis and uveitis) syndrome, were normal.

Fractions of complement (C3, C4), autoantibodies (ANA, antiDNA, pANCA, cANCA, ENA), serology for HIV, HCV, HBV and toxoplasmosis were all in normal range.

A steroid treatment was started, and, since the age of onset was not typical for a minimal change disease, a kidney biopsy was performed, the results of which showed a focal segmental glomerulosclerosis (FSGS).

Due to the persistence of both peripheral oedema and significant proteinuria after 1 month of treatment, an immunosuppressive therapy with tacrolimus was added. Over the following weeks not only no significant improvement was observed but the patient also developed long-lasting fever (15 days) and arthralgia.

**Fig. 1:**
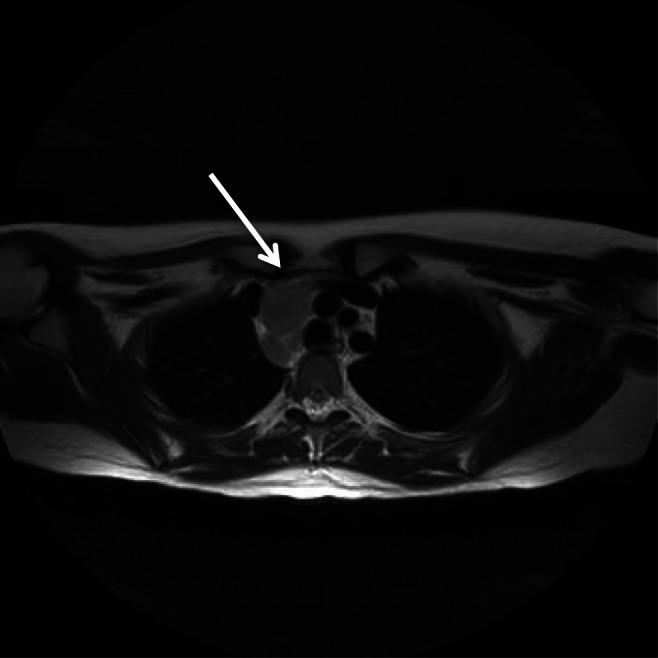
Magnetic resonance imaging (MRI) sequence

In view of the age of onset, the atypical course of the disease and the symptoms developed while on immune-suppressive therapy a workup for secondary glomerulopathies was repeated: blood tests, blood cultures, viral markers, chest x-ray and abdominal ultrasound were all normal except for increased CRP levels (21.1 mg/dL) and erythrocyte sedimentation rate (ESR) (56 mm/h). Due to the persistence of the fever and the increased inflammatory parameters in a patient with otherwise normal laboratory tests, ultrasonography and x-ray investigations, a total body magnetic resonance imaging (MRI) was performed to further investigate (Fig. 1).

## Questions

1. What is the most likely diagnosis?
2. Which are the clinical features suggestive of this condition?
3. What are the treatment and the prognosis of the disease?

